# The spectral composition of a white light influences its attractiveness to *Culex pipiens* mosquitoes

**DOI:** 10.1002/ece3.9714

**Published:** 2023-01-06

**Authors:** Roksana Wilson, Christopher E. C. Cooper, Rochelle J. Meah, Andrew Wakefield, Nicholas W. Roberts, Gareth Jones

**Affiliations:** ^1^ School of Biological Sciences University of Bristol Bristol UK; ^2^ School of Computer Science, Electrical and Electronic Engineering, and Engineering Maths University of Bristol Bristol UK

**Keywords:** Diptera, light attraction, metamerism, phototaxis, spectral wavelength preferences

## Abstract

Insect attraction to artificial light can potentially facilitate disease transmission by increasing contact between humans and vectors. Previous research has identified specific wavelength bands, such as yellow and red, that are unattractive to biting flies. However, narrow‐band, non‐white lights are unsuitable for home lighting use as their very poor color rendering is often considered aesthetically undesirable. The creation of a white light that is unattractive to insects has so far remained elusive. White light can be created by combining a number of narrow‐band light‐emitting diodes (LEDs). Through choice chamber experiments on *Culex pipiens* (*Cx. pipiens*) mosquitoes, we examine whether combining specific wavelength bands has an additive, subtractive or synergistic effect on insect attraction. We show that a white light created by combining narrow‐band red, green and blue (RGB) LEDs is less attractive to *Cx. pipiens* than a broad‐spectrum white light; and that a white light created by combining narrow‐band blue and yellow LEDs is more attractive than a broad‐spectrum white light. White light produced by RGB combinations could therefore serve as a safer and cheaper light in countries where phototactic vectors and vector‐borne disease are endemic.

## Graphical abstract

*Culex pipiens* mosquitoes do not find all white lights equally attractive. White light created by combining red, green, and blue (RGB) LEDs is less attractive than broad‐spectrum white light.

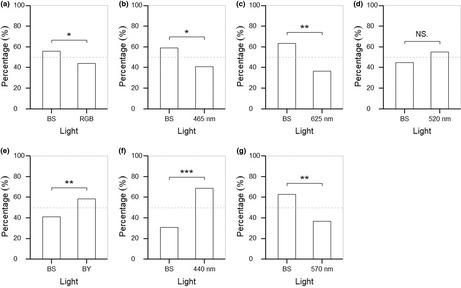

## 1 INTRODUCTION

Urbanization is increasing globally at a rapid rate, and one of the most pervasive signs of an urban space is artificial light at night (ALAN) (Cinzano et al., 2001; Falchi et al., 2016; Kyba et al., 2017). One widely observed consequence of ALAN is the attraction of nocturnal insects to light sources such as streetlights and domestic lights. These insects suffer increased mortality through burning, exhaustion, or predation (Owens et al., 2020; Rich & Longcore, 2013), and it has been suggested that ALAN has contributed to the global decline of insect populations (Owens et al., 2020). Furthermore, the attraction of insects to night lighting represents a public health risk; attraction to light draws nocturnal insect vectors towards human settlements, and diurnal vectors may remain active for longer when under artificial light (Rund et al., 2020). ALAN has been definitively linked to novel modes of Chagas disease transmission and is associated with increased transmission of malaria and leishmaniasis (Barghini & de Medeiros, 2010). Lights may also attract vectors to animal pens where they spread disease amongst livestock and establish reservoirs of infection (Lima et al., 2012). In developing countries, where insect‐transmitted disease is endemic, and urbanization rapid (Zhang, 2016), the development of light sources that minimize insect attraction is of considerable importance.

Not all wavelengths of light are equally attractive to insects. Shorter wavelengths of light (<500 nm) are more attractive to dipterans and as a result, light traps using ultraviolet (<380 nm), blue (450–485 nm), and green (500–565 nm) wavelengths consistently catch more mosquitoes (Culicidae), midges (Ceratopogonidae), and sandflies (Phlebotominae) than those using longer wavelengths (Wilson et al., 2021). However, despite their unattractiveness to biting flies, wavelength bands such as red (625–750 nm) and yellow (565–590 nm) are deemed unsuitable for home illumination as these narrow‐band, non‐white lights have very poor color rendering, which is the ability of a light to show the colors of objects accurately (Barroso et al., 2017). Very poor color rendering is often seen as aesthetically unpleasant (Odabaşioğlu & Olguntürk, 2015), therefore, any lights intended for domestic lighting use would ideally be whiter in color.

Color perception in humans is mediated by the three types of cone photoreceptor in the retina. Each class of cone has a wavelength of maximum sensitivity at 560 ± 5 nm (long wavelength sensitive, LWS), 534 ± 4 nm (medium wavelength sensitive, MWS) and 420 ± 5 nm (short wavelength sensitive, SWS) (Bowmaker & Dartnall, 1980). The excitation and inhibition of two opponent pathways through the outer and inner plexiform layers provide the information that higher processing centers in the brain perceive as color information—or a perception of hue (Solomon & Lennie, 2007; Wyszecki & Stiles, 1982). Importantly, such neural processing leads to metameric color perception, where different spectral power distributions of light lead to the perception of the same color. For example, the perception of white light can be created by combining narrow‐band blue and yellow (BY) or narrow‐band red, green and blue (RGB) spectral profiles. Perceived white light can also be created by broadband emissions across the visible wavelength range. Different metameric combinations therefore have the potential to be viewed as the same by humans, but they may not be equally attractive to insects. This raises the possibility of developing a domestic light that is still perceived as white by humans but is less attractive to insect vectors.

Members of the *Culex pipiens* (*Cx. pipiens*) species complex are found in all known urban and sub‐urban temperate regions (Farajollahi et al., 2011). These mosquitoes are opportunists that primarily feed on avian and mammalian hosts (Brugman et al., 2018), and are consequently considered important bridge vectors for West Nile virus, Rift Valley fever, lymphatic filariasis, and avian malaria (Harbach, 1988; Jung et al., 2021; Kimura et al., 2010). Since the peak of host‐seeking activity in this species occurs in the evening or night time, they are likely to be attracted to ALAN (Veronesi et al., 2012).

The primary objective of this study was to determine whether *Cx. pipiens* mosquitoes found white (as perceived by humans) LED lights with different spectral compositions equally attractive. Broad‐spectrum light was also compared against narrow‐band, non‐white lights to provide a clearer image of how specific wavelength bands interact with each other when combined.

## 2 MATERIALS AND METHODS

### 2.1 Mosquitoes

*Culex pipiens* (Linnaeus, 1758) eggs were provided by the Pirbright Institute, Woking, UK, and reared following the supplier's instructions (Gerberg et al., 1994). The mosquitoes were maintained in a controlled insectary at 25 ± 1°C, 55%–60% RH under a 16:8 h light: dark cycle. Dark hours were between 11:00 and 18:00 h. Larval mosquitoes were housed in a 22 × 22 × 13 cm plastic container filled with 3.5 L of tap water inside a 31 × 28 × 31 cm mesh cage. Larvae were given three guinea pig pellets every 2 days and reached adulthood after 17 days. Adult mosquitoes were transferred to a separate 31 × 28 × 31 cm mesh cage via a pooter and provided with cotton pads soaked in 10% sugar solution, which was replenished daily. They were not blood‐fed. Choice chamber assays were performed on adult mosquitoes that were between one and 7 days post‐eclosion and both males and females were used. Mosquitoes were reared and treated according to ethical rules and regulations.

### 2.2 Lighting

Eight lighting treatments were used in total. These consisted of three white lights: Broad‐spectrum (BS); Red Green Blue (RGB); and Blue Yellow (BY); as well as five narrowband LEDs (FWHM—XXnm) 440, 465, 520, 570, and 625 nm.

**FIGURE 1:**
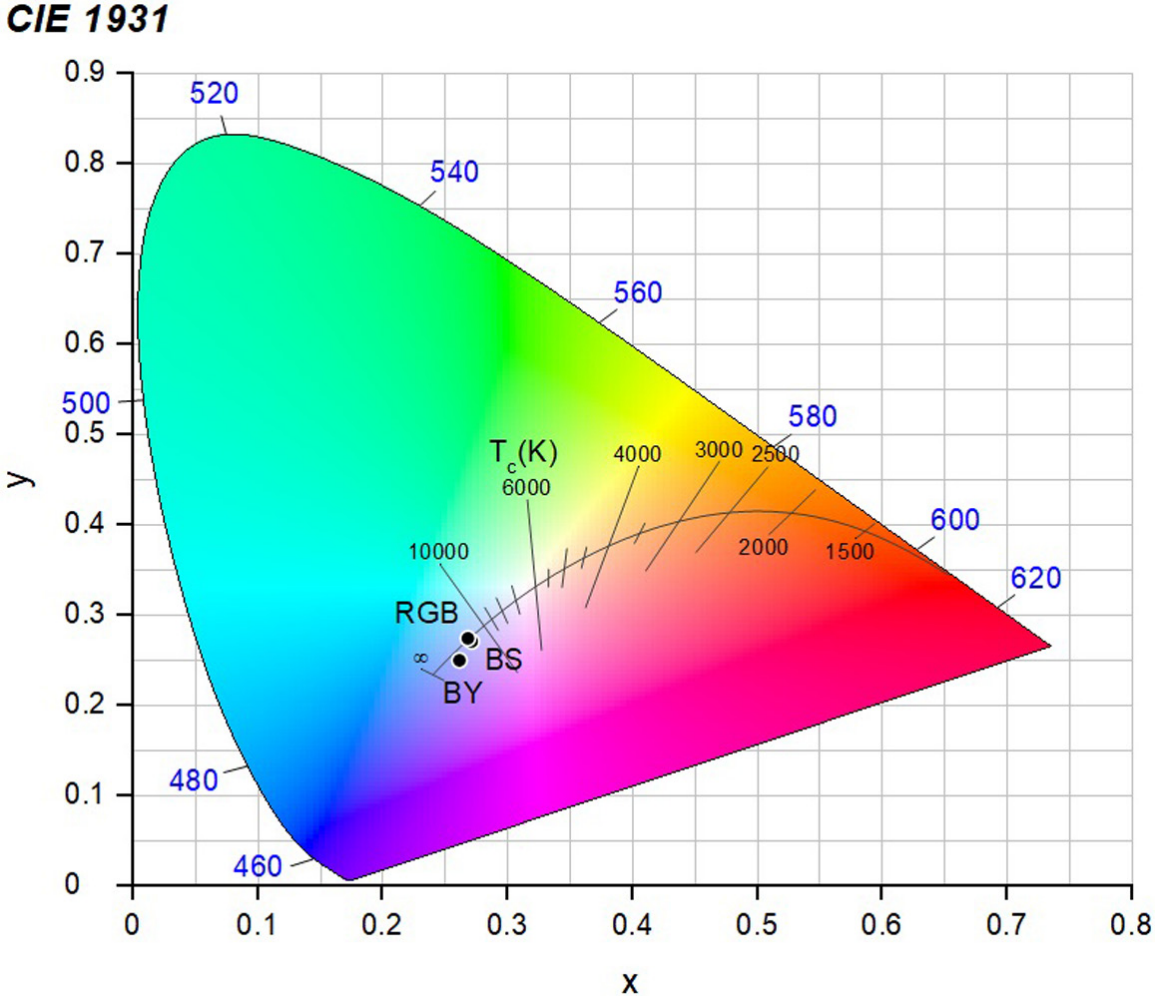
CIE 1931 xy chromaticity diagram for three white lamps: BS, RGB, and BY, used in behavioral experiments. The x and y coordinates indicate the chromaticity. The black curve in the Center is the Plankian locus (or black body locus) which shows the color temperature (T_c_) of the light measured in kelvin (K)—Which goes from a yellow‐tinted (warm) white at lower kelvin (below 3300 K), to a blue‐tinted (cool) white at higher kelvin (above 5300 K) British Standards Institution. (2011).

These narrowband LEDs were used in different combinations to construct the three white lamps, each surrounded by a white, plastic casing, and connected to an Arduino UNO microcontroller. The first lamp used a single broad‐spectrum LED to produce BS white light. The second lamp used one blue (465 nm) LED, one green (520 nm) LED, and two red (625 nm) LEDs to produce RGB white light. The third lamp used one blue (440 nm) LED, and seven yellow (570 nm) LEDs to produce BY white light. Strips of 251 Quarter White Diffusion filter (LEEFilters) were attached to the inside of the plastic casing to blend the light emitted from the LEDs. All three white lamps were a cool white (Figure 1).

**FIGURE 2:**
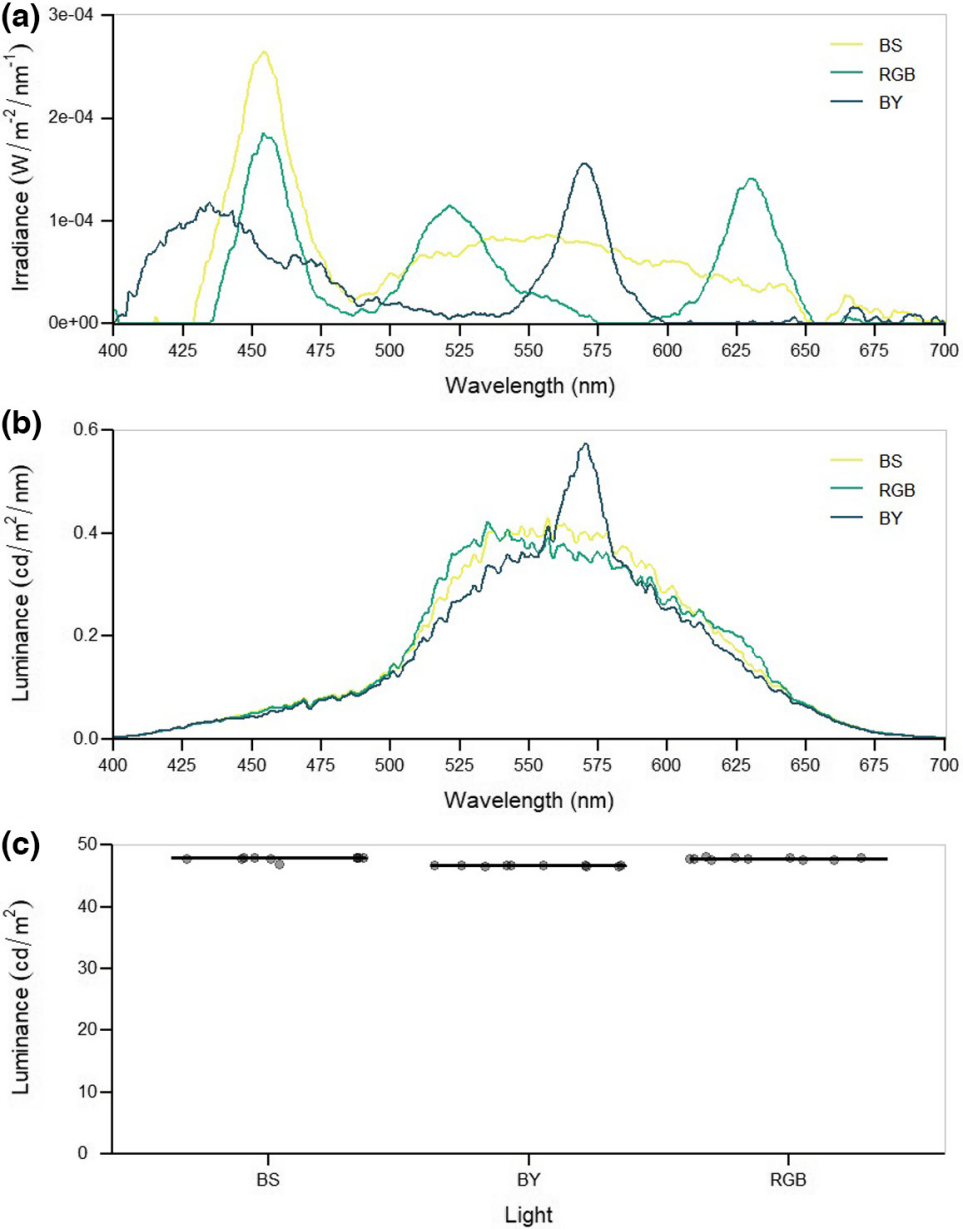
Irradiance and luminance of three white lamps: BS, RGB, and BY, used in behavioral experiments. Spectral irradiance (a) and spectral luminance (b) were measured for the range of wavelengths relevant for human vision. Boxplots show luminance values (c)—which are a mean of 10 measurements.

Measurements of spectral irradiance (Figure 2a), spectral luminance (Figure 2b) and luminance (Figure 2c) were taken with a NIST calibrated StellarNet radiometer using SpectraWiz software v5.33 (https://www.stellarnet.us/). The mean luminance values—an average of 10 measurements—were 47.86 cd/m^2^ (BS), 47.82 cd/m^2^ (RGB) and 46.70 cd/m^2^ (BY). Therefore, it is likely that the lights appear equally bright to humans. The Weber fraction for brightness in humans is estimated to be between 0.08 and 0.14 (Cornsweet & Pinsker, 1965; Griebel & Schmid, 1997; Schiffman, 2001)—meaning two lights would need to have an intensity difference of 8%–14% for one to appear noticeably brighter.

Single narrowband lights were created by turning off the other colored LEDs in each white lamp. For example, to assess the attractiveness of 570 nm light, the 440 nm LED in the BY light was turned off. The non‐white lights were therefore less luminant than the white lights. For example, the sum of the 570 and 440 nm LEDs was 46.70 cd/m^2^, and the luminance of the 570 nm LEDs was the remaining luminance after the 440 nm LED was turned off.

### 2.3 Choice chamber

**FIGURE 3:**
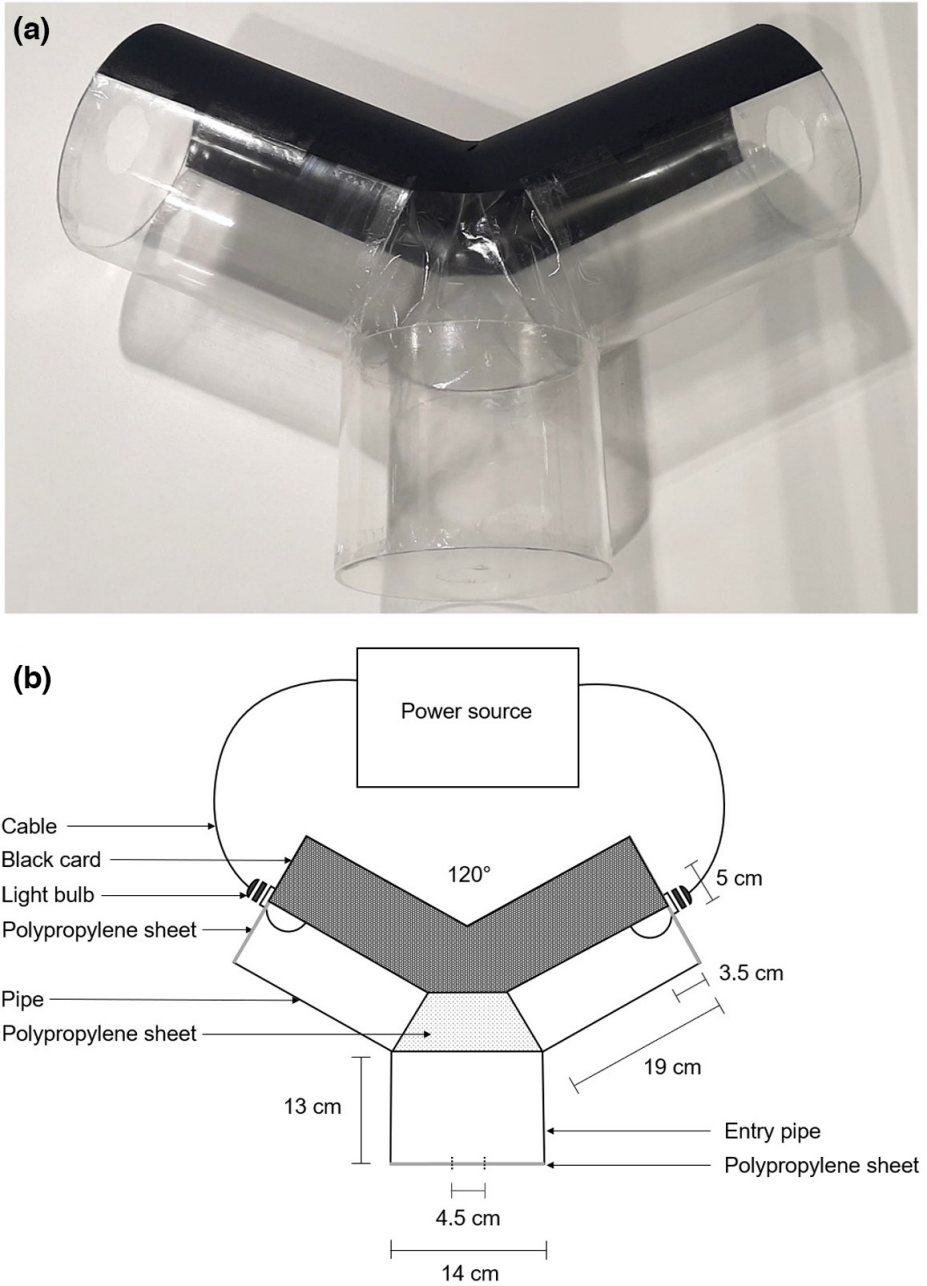
Photo and schematic illustrating the mosquito choice chamber used in behavioral experiments. The choice chamber (a) was constructed from clear, acrylic pipes and partially covered with black card to prevent light from mixing in the arms. Light bulbs were inserted into the two arms (b) and adult *Culex pipiens* mosquitoes were given a choice between two lights. Mosquitoes were placed, one at a time, into the entry pipe of the choice chamber and their immediate resting place recorded. Mosquitoes were only ever used once.

The main body of the choice chamber was constructed from three clear, acrylic pipes: an entry pipe measuring 14 × 13 cm, and two arm pipes measuring 14 ×19 cm (Figure 3). The acrylic used was UV (<400 nm) blocking however, as none of the lights used in this study contained UV light their spectral composition would not have been affected by this material. The end of each pipe, and the gap between them, was covered with a polypropylene sheet. The 5 cm wide lamps were inserted 3.5 cm deep into the arms of the choice chamber through holes in the polypropylene sheets. A 4.5 cm wide hole in the sheet covering the entry pipe allowed mosquitoes to be released into the chamber. The back wall of the choice chamber was covered with black card so light from one arm did not shine into the other arm.

### 2.4 Choice chamber assays

The choice chamber assays were divided into three parts. Part one involved only perceived white light: BS light was compared against either RGB light (*N* = 350) or BY light (*N* = 300). For part two, BS light was compared against the narrowband LEDs that, when combined, made BY light: adult mosquitoes were given a choice between BS light and either 440 nm light (*N* = 100), or 570 nm light (*N* = 100). For part three, BS light was compared against the narrowband LEDs that made up the RGB light: mosquitoes chose between BS light and either 465, 520, or 625 nm light (*N* = 127, 120 and 120, respectively).

Choice chamber assays were performed in a controlled room at 25 ± 1°C, 80% ± 5% RH between 10:00 and 18:00. The lamps inside the choice chamber were the only light source.

A pilot test, where mosquitoes were released, one at a time, into the chamber and then observed for 10 min, revealed that the insects made their ‘choice’ immediately and did not change location unless disturbed. Mosquitoes that failed to leave the entry pipe, remained in the entry pipe for the entire 10‐min period. Therefore, in this study, mosquitoes were released, one at a time, into the choice chamber, and their immediate resting place recorded. The mosquito was then immediately removed from the chamber via a pooter, and the test repeated with another mosquito. Each mosquito was only used once. Mosquitoes that did not leave the entry pipe (either deliberately or because they had been injured during the transfer process) were not included in the analysis. The two lamps in the arms of the choice chamber were initially swapped from left to right after every mosquito. This was done to control for any bias mosquitoes may have towards one side of the chamber. However, this was later changed to after every five mosquitoes as the microcontrollers could be damaged by frequent plugging/unplugging. The choice chamber was also flipped upside down each day, again to control for potential directional preference bias.

### 2.5 Statistical analysis

The Null hypothesis, that *Cx. pipiens* mosquitoes find all lights equally attractive, was analyzed in R v4.0.4 (http://www.R‐project.org/) using a Chi‐square goodness of fit test. *p*‐values were corrected for multiple testing using the sequential Benjamini‐Hochberg procedure with a false discovery rate (FDR) of 0.05 (Benjamini & Hochberg, 1995).

## 3 RESULTS

**FIGURE 4:**
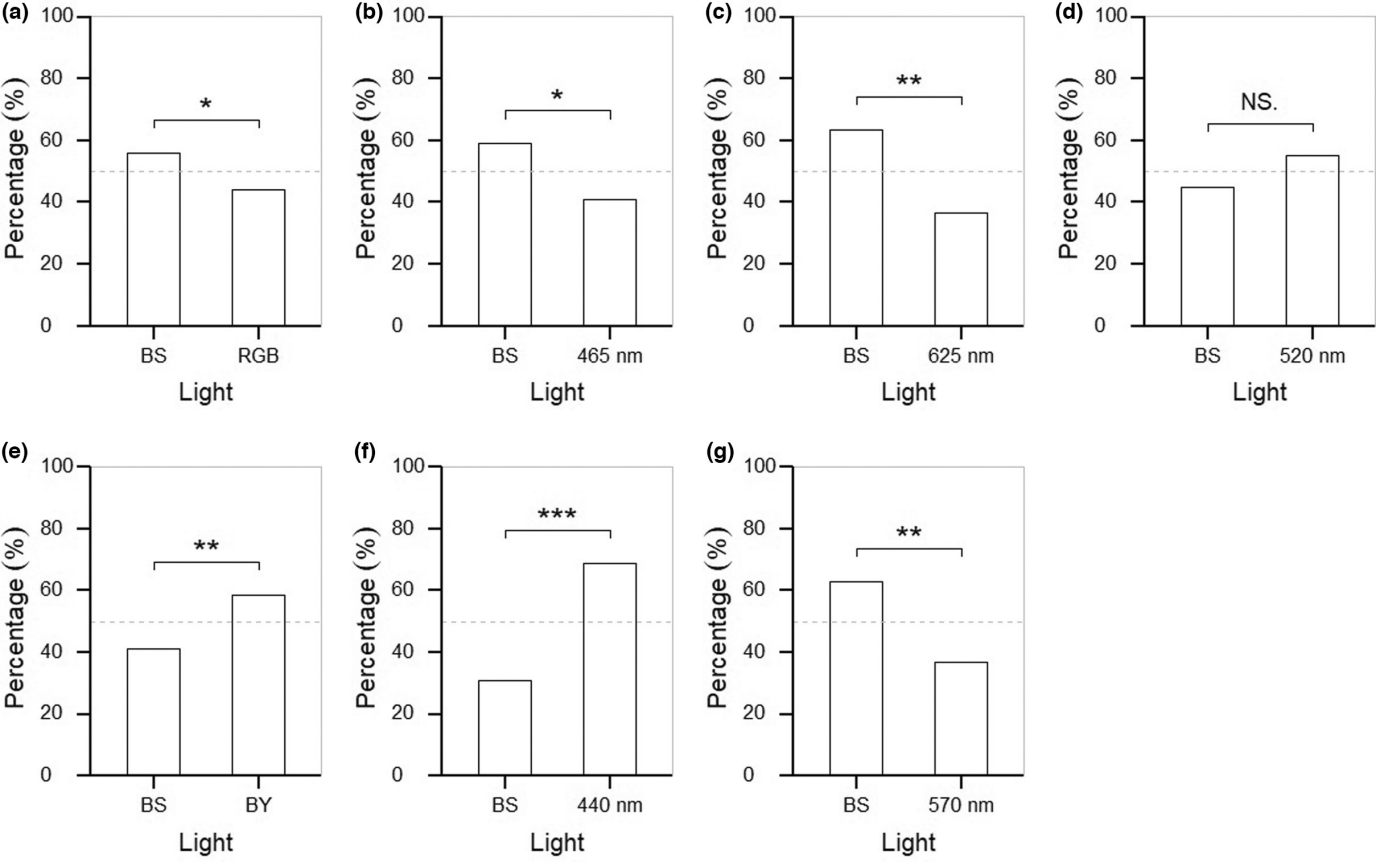
Bar charts showing the percentage of mosquitoes attracted to each lamp in choice chamber assays. BS light was compared against (a) RGB light (*N* = 350), (b) 465 nm light (*N* = 127), (c) 625 nm light (*N* = 120), (d) 520 nm light (*N* = 120), (e) BY light (*N* = 300), (f) 440 nm light (*N* = 100), and (g) 570 nm light (*N* = 100). Results were analyzed using a chi‐squared goodness of fit test and corrected for multiple comparisons using the Benjamini‐Hochberg procedure (FDR = 0.05). Asterisks indicate a significant difference: ^NS^ *p* > .05, *p < .05, **p < .01 and ***p < .001.

In total, 1252 mosquitoes were tested—35 of which did not leave the entry pipe and were therefore excluded from the analysis. The BS light attracted significantly more mosquitoes than the RGB, 465, and 625 nm lights; and significantly fewer mosquitoes than the BY, 440, and 570 nm lights. The 520 nm light attracted a higher proportion of mosquitoes than the BS light, however, this difference was not significant (Table 1; Figure 4).

**TABLE 1:** Chi‐square goodness of fit test, with a Benjamini‐Hochberg correction, for the choice chamber assays.

Test pair	Chi‐square statistic	Degrees of freedom	Sample size	*p*‐Value	B‐H CV
BS vs. 440 nm	14.44	1	100	**<.001**	0.007
BS vs. BY	9.01	1	300	**.003**	0.014
BS vs. 625 nm	8.53	1	120	**.003**	0.021
BS vs. 570 nm	6.76	1	100	**.009**	0.029
BS vs. RGB	5.04	1	350	**.025**	0.036
BS vs. 465 nm	4.17	1	127	**.041**	0.043
BS vs. 520 nm	1.20	1	120	.273	0.050

*Note*: Test pairs with their corresponding chi‐square statistic, degrees of freedom, sample size, *p*‐value, and Benjamini‐Hochberg critical value (B‐H CV). The B‐H CV was calculated based on the Benjamini‐Hochberg procedure with the FDR set to 0.05. Significant results are highlighted in bold.

There was no association between direction and light preference for any light.

## 4 DISCUSSION

In choice chamber assays comparing white lights, the BS light attracted significantly fewer *Cx. pipiens* mosquitoes than BY light, and significantly more mosquitoes than RGB light (Figures 4a,e). While color temperature and brightness of all the white lights were similar for human vision, differences in spectral content and brightness to an insect visual system resulted in a different perceived attractiveness.

A number of studies have found that warmer‐toned, lower color temperature, white LEDs attract fewer insects than cool‐toned, higher color temperature, white LEDs—the latter of which have more power between 400 and 500 nm (Deichmann et al., 2021; Eisenbeis & Eick, 2011; Longcore et al., 2015). In this study, the BY light is shorter wavelength shifted compared with the BS or RGB lights and has more power below 430 nm, and the RGB light contains less power between 430 and 480 nm than the BS light (Figure 2a). This likely explains the pattern of attractiveness observed here. Reducing the amount of short wavelength light in white LEDs may result in fewer insects approaching houses. It may also benefit other wildlife, such as migrating birds and turtle hatchlings, who are also attracted to short‐wavelength bands and may become lost, injured or killed upon approaching lights (Rivas et al., 2015; Zhao et al., 2020). Having a lower ratio of short: high wavelength bands may also have the added benefit of reducing human sleep disorders and eye disease, as short wavelength light has a larger effect on circadian rhythms and is more damaging to the retina (Grimm et al., 2001; Lockley et al., 2003).

However, other studies have found that the color temperature of a white LED does not significantly influence its attractiveness (Pawson & Bader, 2014; Spoelstra et al., 2015; Wakefield et al., 2016). Resolving the cause of these conflicting results is difficult, in part due to differences in experimental design, potential confounds in perceived brightness, habitats surveyed, and species tested. Other factors such as insect sex and age may also affect light attraction.

In assays comparing white and narrowband colored LEDs, the 440 nm LED on its own attracted a higher proportion of mosquitoes (69%) than the BY (440 nm LED + 570 nm LEDs) light did (59%) when both were compared against BS light (Figures 4e,f). In essence, the addition of 570 nm light to 440 nm light produced a less attractive light despite the increase in luminance. Similarly, the 520 nm LED attracted a higher proportion of mosquitoes (55%) than the more luminous RGB (520 nm LED + 465 nm LED + 625 nm LEDs) light did (44%) when both were compared against BS light (Figures 4a,d). ‘Spectral purity’ likely plays a role in insect attraction, and future studies should directly test combination lights (e.g., RGB) against their component LEDs (e.g., green) to determine whether less intense narrow‐band lights are more attractive than brighter white lights. While it is possible that the mixing of wavelength bands changed the hue of the light to one the mosquitoes found less attractive, a preference for one spectral composition over another is not evidence of color vision. This phenomenon can be better characterized as a wavelength‐specific response: positive phototaxis is triggered by short and medium wavelength bands (<500 nm) and inhibited by long wavelength bands (>500 nm) (Yang et al., 2004).

True color vision is the ability of an organism to discriminate between lights of different spectral compositions (hues) independently of their intensity (Gao et al., 2008). It requires, at minimum, two photoreceptor types that are sensitive to different regions of the electromagnetic spectrum and a neural framework that can compare the output of these receptor types (Kelber et al., 2003). As with other dipterans, mosquito ommatidia contain eight photoreceptor cells (R1–R8) (Hu et al., 2009; Land et al., 1999). The R1–R6 cells mediate achromatic sensitivity in flies (Sanes & Zipursky, 2010), and in crepuscular and nocturnal mosquitoes, the rhabdomeres of these cells are all connected to form a ring‐like structure known as a fused rhabdom. This fused rhabdom increases light sensitivity, at the expense of visual acuity, and is advantageous in a low light environment (Hu et al., 2009). The R7 and R8 cells are involved in fly color vision and typically follow a one photoreceptor/one rhodopsin scheme (Hu et al., 2009; Sanes & Zipursky, 2010). In mosquitoes, however, the R7 and R8 cells have a complex pattern of short wave (<400 nm), mid‐wave (400–500 nm), and/or long wave (>500 nm) opsin co‐expression depending on the eye region (Hu et al., 2009, 2011, 2014). Opsin co‐expression increases photon capture, and therefore light sensitivity, but comes at the expense of wavelength discrimination. It is therefore unknown whether the R7 and R8 cells mediate color vision in mosquitoes as they do in other dipterans.

Whether an animal possesses color vision is normally assessed using behavioral experiments: an animal is trained to associate a color stimulus with sugar solution and is then made to choose between the color stimulus and multiple shades of gray (Frisch, 1914). If the animal is only using achromatic information, then it would be unable to distinguish between the color stimulus and at least one shade of gray. These types of experiments have been performed on only a few dipteran species: the bee fly *Bombylius fuliginosus* (Knoll, 1926), the hoverfly *Eristalis tenax* (Ilse, 1949), and the blowfly *Lucilia cuprina* (Fukushi, 1990), none of which are biting flies. Mosquitoes can also be trained to associate certain visual cues with a sugar solution (Bernáth et al., 2016), which suggests that similar color vision experiments can be performed on mosquitoes.

The difference in attractiveness between the BS and RGB lights was 24% and this could potentially translate into hundreds fewer insects if many houses over a wide area switch to RGB light. However, there are technical barriers that must be overcome for color‐mixed lights to see widespread commercial usage. Firstly, the uniform mixing of colors so that the light emitted from the bulb appears white when viewed from all angles is difficult to achieve. Temperature, time, and current all change the brightness and spectral irradiance of an LED, exacerbating this color mixing problem. As white light is created by mixing specific wavelengths in specific ratios, a change in the intensity or dominant wavelength of one LED will considerably affect how white the light appears. For example, an RGB light may become greener over time as the red LED degrades faster than the blue and green LEDs. Barriers to the adoption of color‐mixed lights and the potential solutions to these problems are outlined in Muthu et al. (2002).

As the white lights used in this study were designed to be used as houselights, the attractiveness of RGB light compared with BS and BY light should now be tested in the field. Wild mosquitoes may not have the same ‘preferences’ as laboratory‐bred and reared mosquitoes, and the reflection of light against the surfaces of the choice chamber may also have an effect on the attractiveness of the lights (Hoel et al., 2007). Phototaxis is also, in part, controlled by the circadian clock and the unusually long day: night cycle used in this study may affect insect behavior (Baik et al., 2020). The way the mosquitoes were introduced to the lights (via pooter‐transfer) may also influence their behavior: the mosquitoes could be exhibiting an escape response and might not have the same ‘preferences’ when under more relaxed circumstances. Using the lights in the field would also reveal whether the pattern of attractiveness seen here (BY > BS > RGB) is found in other insect groups.

How these lights perform against darkness must also be examined, as the ‘perfect’ house light from a vector control perspective would be one that attracts as many, or fewer insects than darkness. Additionally, combining blue and yellow, or red, green, and blue LEDs are not the only ways of producing white. Other color mixes may be less attractive to insects than RGB light, and a better understanding of how color opponency works in dipterans may allow us to predict which mixed lights would be minimally attractive. With further work, the mixing of light from a small number of colored LEDs has the potential to mitigate public health risks caused by vector attraction and result in fewer disturbances to nocturnal wildlife.

## AUTHOR CONTRIBUTIONS

**Roksana Wilson:** Conceptualization (lead); data curation (lead); formal analysis (lead); investigation (lead); methodology (equal); project administration (lead); resources (lead); software (equal); validation (lead); visualization (lead); writing – original draft (lead); writing – review and editing (lead). **Christopher E. C. Cooper:** Methodology (equal); software (equal); validation (supporting). **Rochelle J. Meah:** Software (supporting); validation (supporting). **Andrew Wakefield:** Supervision (equal); writing – review and editing (supporting). **Nicholas W. Roberts:** Software (supporting); supervision (equal); validation (supporting); writing – review and editing (supporting). **Gareth Jones:** Funding acquisition (lead); resources (supporting); supervision (equal); writing – review and editing (supporting).

## FUNDING INFORMATION

RW was supported by a Natural Environment Research Council Industrial Collaborative Awards in Science and Engineering PhD studentship partnered with Integral LED, UK under grant code NE/R008701/1. Funders did not contribute to the conception, writing or editing of the manuscript or the decision to publish.

## CONFLICT OF INTEREST

No competing interests declared.

## Data Availability

The data that supports the findings of this study is openly available from the Dryad Digital Repository (https://doi.org/10.5061/dryad.pnvx0k6s0).
